# Antibody responses to the full-length VAR2CSA and its DBL domains in Cameroonian children and teenagers

**DOI:** 10.1186/s12936-016-1585-y

**Published:** 2016-11-04

**Authors:** Barriere A. Y. Fodjo, Njika Atemnkeng, Livo Esemu, Emile K. Yuosembom, Isabella A. Quakyi, Viviane H. M. Tchinda, Joseph Smith, Ali Salanti, Jude Bigoga, Diane W. Taylor, Rose G. F. Leke, Anna Babakhanyan

**Affiliations:** 1 Department of Biochemistry, Faculty of Medicine and Biomedical Research, Biotechnology Centre, University of Yaoundé 1, Yaounde, Cameroon; 2 School of Public Health, College of Health Sciences, University of Ghana, Legon, Ghana; 3 Seattle Biomedical Research Institute, Seattle, WA 98109 USA; 4 Department of Immunology and Microbiology, Centre for Medical Parasitology, University of Copenhagen, Copenhagen, Denmark; 5 Department of Tropical Medicine, Medical Microbiology and Pharmacology, University of Hawaii at Manoa, John A Burns School of Medicine, 651 Ilalo Street, BSB 320, Honolulu, Hawaii 96813 USA

**Keywords:** Malaria, VAR2CSA, Antibody, Children

## Abstract

**Background:**

Antigenic variation of *Plasmodium falciparum* erythrocyte membrane protein 1 is a key parasite mechanism for immune evasion and parasite survival. It is assumed that the number of parasites expressing the same *var* gene must reach high enough numbers before the host can produce detectable levels of antibodies (Ab) to the variant. VAR2CSA is a protein coded for by one of 60 *var* genes that is expressed on the surface of infected erythrocytes (IE) and mediates IE binding to the placenta. The idea that Ab to VAR2CSA are pregnancy-associated was challenged when VAR2CSA-specific Ab were reported in children and men. However, the frequency and conditions under which Ab to VAR2CSA are produced outside pregnancy is unclear. This study sought to determine frequency, specificity and level of Ab to VAR2CSA produced in children and whether children with hyperparasitaemia and severe malaria are more likely to produce Ab to VAR2CSA compared to healthy children.

**Methods:**

Antibody responses to a panel of recombinant proteins consisting of multiple VAR2CSA Duffy-binding-like domains (DBL) and full-length VAR2CSA (FV2) were characterized in 193 1–15 year old children from rural Cameroonian villages and 160 children with severe malaria from the city.

**Results:**

Low Ab levels to VAR2CSA were detected in children; however, Ab levels to FV2 in teenagers were rare. Children preferentially recognized DBL2 (56–70%) and DBL4 (50–60%), while multigravidae produced high levels of IgG to DBL3, DBL5 and FV2. Sixty-seven percent of teenage girls (n = 16/24) recognized ID1–ID2a region of VAR2CSA. Children with severe forms of malaria had significantly higher IgG to merozoite antigens (all p < 0.05), but not to VAR2CSA (all p > 0.05) when compared to the healthy children.

**Conclusion:**

The study suggests that children, including teenage girls acquire Ab to VAR2CSA domains and FV2, but Ab levels are much lower than those needed to protect women from placental infections and repertoire of Ab responses to DBL domains is different from those in pregnant women. Interestingly, children with severe malaria did not have higher Ab levels to VAR2CSA compared to healthy children.

**Electronic supplementary material:**

The online version of this article (doi:10.1186/s12936-016-1585-y) contains supplementary material, which is available to authorized users.

## Background

*Plasmodium falciparum* remodels the host erythrocyte membrane upon invasion to promote parasite survival and immune evasion [1–5]. *Plasmodium falciparum* erythrocyte membrane protein 1 family (PfEMP1) is encoded by the *var* multigene family enable infected erythrocytes (IE) to become adhesive and facilitates IE binding to the vasculature, as an immune evasion mechanism [6, 7]. Each parasite genome contains about 60 different *var* genes with high sequence diversity; however, at any given time, only one *var* gene is expressed within a single IE [8], a process regulated at the level of transcription initiation [9–11]. Placental *P. falciparum* parasites primarily express only *var2csa* [12–14], which appears to be regulated both at the transcription level and at translation initiation [15, 16]. In pregnant women, the adhesion ligand VAR2CSA binds to chondroitin sulfate A (CSA) mainly in the placental intervillous space and on syncytiotrophoblasts lining the intervillous space of the placenta [5, 12, 17, 18].

VAR2CSA is a large transmembrane protein [19] that is relatively conserved for the *var* gene family [20]. It is composed of six Duffy-Binding-Like domains (DBL domains 1–6), interspersed by inter-domain regions (ID). Recently, the minimal sequence of VAR2CSA required for binding to CSA, ID1–ID2a, which spans DBL2 was identified [21, 22]. As a result of IE binding to CSA, IE accumulate at the maternal-fetal interface causing placental malaria (PM). Pathology resulting from PM increases the risk of maternal anemia and poor pregnancy outcomes [23, 24]. In malaria endemic areas, pregnant women produce antibodies (Ab) to VAR2CSA over successive pregnancies [25] that inhibit the binding of IE to CSA in vitro [26, 27], reduce maternal anaemia [28], and improve pregnancy outcome [25, 29, 30]. VAR2CSA-based recombinant subunit vaccine candidates are currently under clinical evaluation [31, 32].

Ab to VAR2CSA are thought to be pregnancy specific; however, studies showed that they can also be detected in non-pregnant individuals including men and children [33–37]. It is assumed the number of IE expressing the same *var* gene must reach a high enough levels before the host can begin producing a detectable Ab response to each variant. Previous studies suggest that expression of *var2csa* in non-pregnant individuals results in sufficient exposure to the VAR2CSA to induce an Ab response [34, 35], with biologically relevant levels confirmed by using the adhesion inhibition assay [33], but cross-reactive since they can be induced also by *Plasmodium vivax* [35]. Ab to VAR2CSA among non-pregnant individuals from sub-Saharan Africa are generally low [33, 34, 36], with exception to ID1–ID2a [36], while medium–high levels were observed in Colombians children and men [35]. The frequency and conditions under which Ab to VAR2CSA are produced in children and males are unclear.

Previous studies measured Ab to the surface of CSA-binding IE [33] or to a limited number of recombinant VAR2CSA domains [34–36] and were either limited to children below 6 months [34] (who may still carry placentally-transferred maternal IgG) or in undefined age groups [33, 35]. The current study sought to determine (1) when maternal IgG to VAR2CSA and its domains decline in neonates in the absence of exposure to *var2csa* expressing parasites, (2) whether individuals acquire antibodies to VAR2CSA during childhood, and (3) if children who have hyperparasitaemia and develop severe malaria were more likely to produce Ab to VAR2CSA compared to healthy children. Young women who produced Ab to VAR2CSA as children may have an altered immune response upon exposure to VAR2CSA during pregnancy or a future placental malaria vaccine.

## Methods

### Ethical considerations

The use of de-identified archival samples used in the current study was approved by the Committee on Human Studies, University of Hawaii, Manoa (CHS# 22332). The original studies were conducted according to the Helsinki Declaration principles and approved by the National Ethics Committee, Cameroon and the Institutional Review Boards at the University of Hawaii and Georgetown University. Parents or guardians of all children provided written informed consent prior to inclusion of their children into the study.

### Study design

Archival plasma samples from three different studies conducted in Cameroon were used. The first set of archival samples (COHORT 1) was from a longitudinal study conducted between 2008 and 2014 in Ngali II and Ntouessong villages (NCT00593398), as previously described [38]. Pregnant women were recruited during pregnancy and followed monthly; they received intermittent preventive treatment with sulfadoxine-pyrimethamine and insecticide-treated bed nets during pregnancy. Their neonates, who slept under bed nets with their mothers, were followed for the first year of life and plasma samples from cord blood, samples at 4–5 months of age (21–28 weeks) and 9–12 months of age (35–56 weeks) from 38 neonates were included in the study.

The second cross-sectional study (COHORT 2) was conducted between 2001 and 2003 at the Yaoundé Central Hospital pediatric emergency unit, Cameroon [39]. In Yaoundé city, malaria transmission is low with about 13 infectious bite/person/year [40, 41]. Children with blood smear-positive malaria (n = 160) who had uncomplicated malaria (n = 41); cerebral malaria (n = 15); severe malaria anaemia (n = 35); other severe forms (n = 27); as well as healthy children (n = 42) all below five years of age were studied.

The third cohort (COHORT 3) was from a cross-sectional study conducted in rural villages of Ngali II and Ntouessong, Cameroon, in 2012. The study was after the rapid scale-up of malaria intervention strategies through the use of insecticide-treated bed nets for vector control. This resulted in reduction of entomological inoculation rate to 0.34 infectious bites/person/night; however, compared to the Yaoundé city, malaria transmission was higher in the villages. In the 2012 study, 193 children aged 1–15 years old were recruited and evaluated in the Ngali II and Ntouessong villages. Finally, plasma from 11 North American adults never exposed to malaria and 17 pregnant multigravidae Cameroonian women with high IgG levels to VAR2CSA FCR3 strain were used as negative and positive controls, respectively.

### Diagnosis of malaria and definitions

*Plasmodium falciparum* infections in peripheral and cord blood were detected by microscopy as previously described [40]. Uncomplicated malaria (UM) was defined as clinical symptoms of malaria, a positive peripheral blood smear parasitaemia, but with no complications. Cerebral malaria (CM) was defined as a Blantyre coma score of <3 (persisting for more than 30 min after effective treatment of hypoglycemia or seizures) in a child with *P. falciparum* parasitaemia and no other apparent cause of coma. Severe malaria anemia (SMA) defined according to WHO’s definition criteria: haemoglobin level <5.0 g/dL (or haematocrit <15%) combined with a malaria parasitaemia. Others severe forms (OSF) of malaria were defined as complications other than the previous ones cited, mainly cases of seizures, prostration, hypoglycaemia, etc., with a positive peripheral blood smear. Healthy children (HC) were defined as age matched healthy children with a negative peripheral blood smear.

### Recombinant malaria proteins

Recombinant VAR2CSA DBL domains used in this study were described previously and include: DBL1 3D7, DBL1 7G8, ID1–ID2a FCR3, DBL2 FCR3, DBL3 FCR3, DBL3 7G8, DBL3 A4/FCR3, DBL4 FCR3, DBL4 7G8, DBL5 FCR3, DBL5 3D7, DBL5 7G8, DBL6 FCR3, and full-length VAR2CSA (FV2) FCR3 [36, 37]. Other non-pregnancy specific antigens included were recombinant AMA-1 (3D7 strain) expressed in yeast provided by the Malaria Vaccine Development Branch (MVDB), NIH; recombinant EBA-175 RII expressed in yeast obtained from Science Applications International Corp., Frederick, MD; recombinant MSP-1 42 (3D7 strain) expressed in *Escherichia coli* (MVDB); recombinant MSP-2 (FC27 strain) (MVDB); recombinant MSP-3 C-terminal region expressed in *E. coli* provided by P. Druihle, Institute Pasteur, Paris, France.

### Coupling of malaria proteins to magnetic beads

VAR2CSA antigens were coupled to the magnetic MagPlex-C microspheres using the coupling protocol described for SeroMap beads [36]. MSP-2 FC27 was coupled at 1 µg per million beads. The rest of the protein concentrations for non-pregnancy specific malaria antigens were described previously [42].

### Measuring IgG using a multi-analyte platform (MAP) assay

IgG to malaria antigens was measured using MAP assay as described before [37]. Modification includes the use of magnetic plate separator instead of vacuum manifold (Luminex, Austin, Texas, Cat# CN-0269-01) and a MAGPIX reader instead of Luminex 100 instrument (EMD Millipore, Billerica, MA). The results were expressed as median fluorescence intensity (MFI). Negative and positive controls were included on each plate consisting of (a) plasma pool of 3 North Americans who had never travelled to malaria endemic areas as negative control and (b) pool of plasma from 7 Cameroonian multigravidae with high Ab levels to FV2 as positive control.

### Statistical analysis

Demographic, clinical and assay variables were summarized with means/medians and standard deviations (or interquartile ranges) for the continuous variables, and frequencies and percentages for categorical variables. Demographic and clinical parameters between three age groups in Table 1 were compared using Chi square trend test for proportions; continuous variables were compared using ANOVA or Kruskal–Wallis test. Proportion of individuals who were seropositive for a given antigen was determined based on mean MFI+2 standard deviation cut-off for 11 North Americans. Mann–Whitney test was used for all Ab level comparisons between any two groups of study participants. All statistical analyses were performed using GraphPad Prism version 6.0; p values less than 0.05 were regarded as statistically significant.

**Table 1:** Characteristics of 193 Cameroonian children in three age groups living in the Ngali II and Ntouessong villages

	1–5 years	6–10 years	11–15 years	p*
Number of children	21	105	67	–
Number of male children (number, %)	10 (48%)	54 (51%)	43 (64%)	0.06
Malaria-positive by blood smears (number, percent)^a^	3 (14%)	64 (61%)	33 (49%)	0.0004
Malaria parasitaemia (parasites per µL, median and 25th, 75th percentile in parentheses)^b^	360 (360, 1320)	320 (170, 780)	360 (120, 590)	0.5
ITN use (number, %)	11 (52%)	70 (67%)	38 (57%)	0.3
Hemoglobin levels in g/dL (mean ± SD)	11 ± 2.3	11.7 ± 1.9	13 ± 1.7	<0.0001
Anemia (number, %)^c^	8 (38%)	47 (45%)	12 (18%)	0.001

* Proportions were compared using Chi square trend test, continuous variables were compared using ANOVA or Kruskal–Wallis test

^a^Blood smears were evaluated by microscopy for presence of *P. falciparum, P. ovale, and P. malariae,* Only *P. falciparum* was detected

^b^Calculated only for smear-positive individuals

^c^Cut-offs for anemia: children 0–5 years < 11 g/dL; 6-10 years < 11.5 g/dL; 11-15 years < 12 g/dL

## Results

### Longevity of passively acquired antibodies to VAR2CSA in Cameroonian neonates

**Fig. 1:**
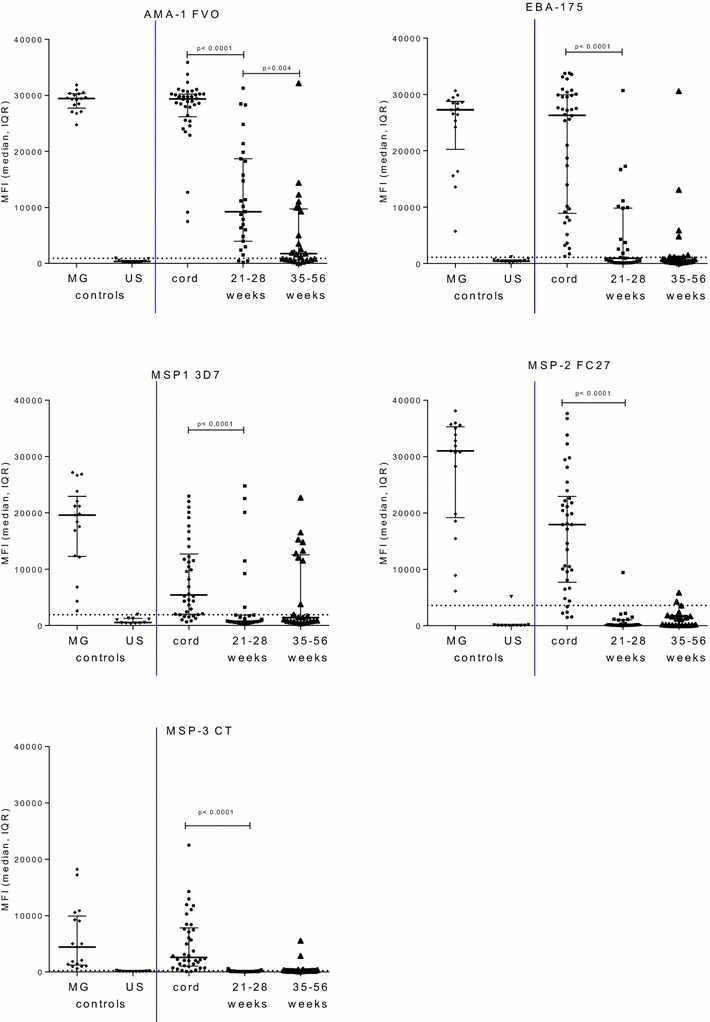
Antibody levels to merozoite antigens in neonates during the first year of life. Antibody levels to 5 merozoite antigens were measured in neonatal samples: n = 38 cord, n = 27 samples from 21 to 28 weeks neonates, and n = 24 samples from 35 to 56 week from neonates residing in Ngali/Ntouessong villages. In addition, 11 North American adults and 17 Cameroonian multigravidae were included as antibody-negative and positive experimental controls. Median and interquartile ranges (IQR) are plotted, antibody levels between two groups were compared using Mann–Whitney test. *Horizontal line* represents cut-off for seropositivity

**Fig. 2:**
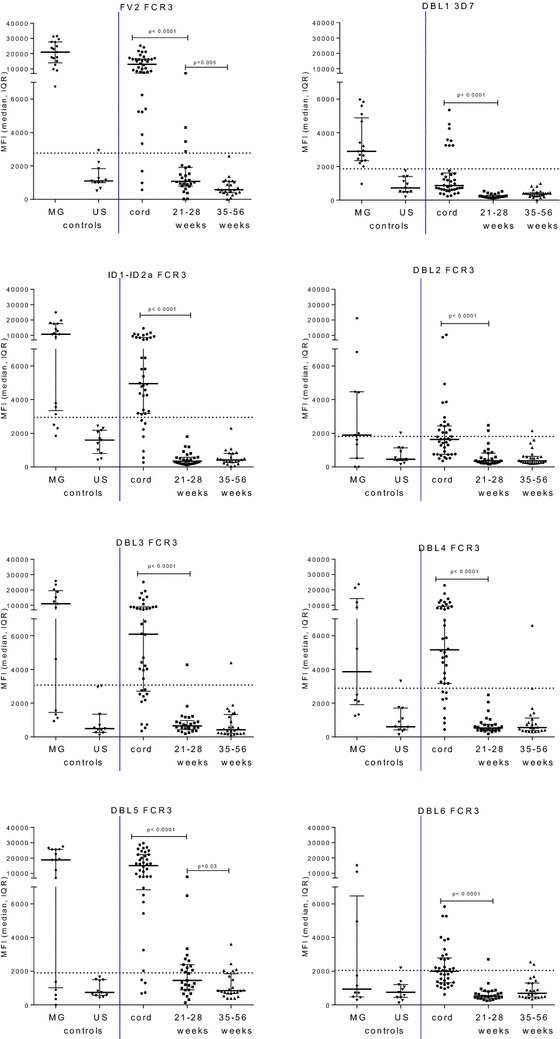
Antibody levels to VAR2CSA domains in neonates during the first year of life. Antibody levels to VAR2CSA DBL domains and full-length protein (FV2) were measured in neonatal samples: n = 38 cord, n = 27 samples from 21 to 28 weeks neonates and n = 24 samples from 35 to 56 week neonates residing in Ngali/Ntouessong villages In addition 11 North American adults and 17 Cameroonian multigravidae were included as antibody-negative and positive experimental controls. Median and interquartile ranges (IQR) are plotted, antibody levels between two groups were compared using Mann–Whitney test.* Horizontal line* represents cut-off for seropositivity

In order to follow the decline of placentally-transferred maternal IgG to malaria antigens, samples collected during the first year of life (COHORT 1) from neonates living in the Ngali II and Ntouessong villages were assessed (Figs. 1 and 2). The prevalence of malaria parasitaemia was negligeable in neonates: 1/27 in 21–28 week old neonates and 3/24 in 36–56 week old neonates, possibly because neonates sleep under bed nets. Ab to merozoite antigens were present in all neonates (Fig. 1). Compared to levels at birth (cord blood), neonatal IgG to MSP1, MSP2 and MSP3 were significantly lower by 21–28 weeks of age (all p values <0.05), while AMA1 and EBA175 IgG levels were significantly reduced by 36–56 weeks (all p values <0.05). At 36–56 week, only a few children had IgG to AMA1 (n = 14), EBA175 (n = 8) and MSP1 (n = 8) (Fig. 1). Similarly, neonatal IgG to FV2 and DBL domains (FCR3 strain) were present at birth in the majority of the neonates (FV2 n = 35, DBL2 n = 14, DBL3 n = 27, DBL4 n = 30, DBL5 n = 34, DBL6 n = 17) (Fig. 2). By 21–28 weeks of age, IgG to all DBL domains were significantly reduced (all p values <0.05) and were below cut-off levels for seropositivity, except for DBL5 and FV2. By 36–56 weeks of age, IgG to DBL5 and FV2 had decreased to levels similar to North American adults (Fig. 2). The same pattern was observed for different allelic variants of VAR2CSA (Additional file 1). Thus, overall IgG to all malarial antigens declined; when high IgG levels were present at birth, IgG to malaria antigens was detected throughout most of the first year of life, whereas lower levels of IgG at birth resulted in absence of IgG by 21–28 weeks of age.

### Cameroonian children living in rural villages acquire antibodies against multiple VAR2CSA domains

The demographic and clinical information of children (COHORT 3) aged 1–5, 6–10 and 11–15 years living in the Ngali II/Ntouessong were summarized in Table 1. The proportion of children using bed nets and being blood smear positive were similar among the different age groups (all p values >0.05, Table 1). The prevalence of malaria parasitaemia in children differed among the age groups, with lowest prevalence in the 1–5 age group (14.3%) and highest in the 6–10 age group (61%), p = 0.0004 (Table 1).

**Fig. 3:**
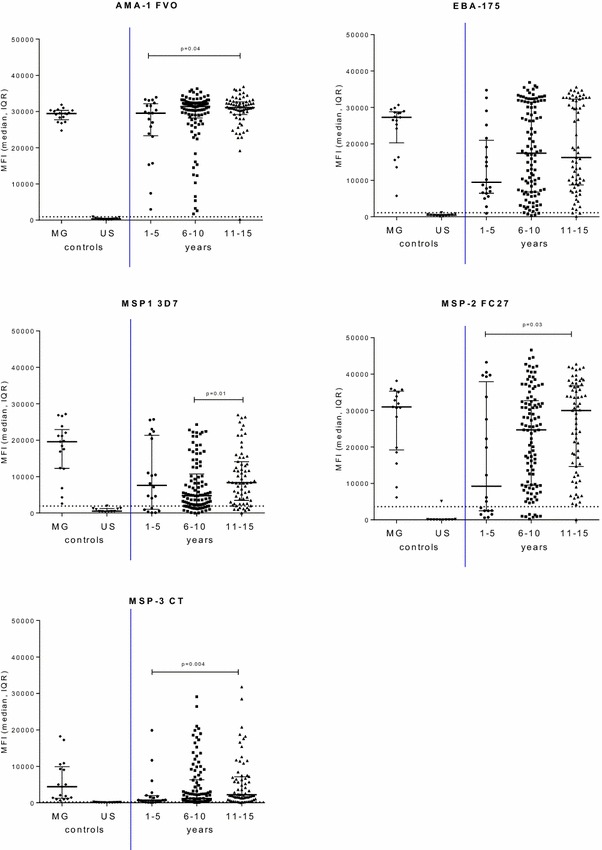
Antibody levels to merozoite antigens in children from Ngali and Ntouessong rural villages. IgG levels to five merozoite antigens were measured in children 0–5 years, 6–11 years and 11–15 years residing in Ngali/Ntouessong. In addition, samples from 11 adult North Americans and 17 Cameroonian multigravidae were also measured as antibody-negative and -positive controls, respectively. Median MFI and Inter-Quartile Range (IQR) are plotted;* dotted line* shows cut-off for positivity. Antibody levels between 2 groups were compared using Mann–Whitney test. *Horizontal line* represents cut-off for seropositivity

**Fig. 4:**
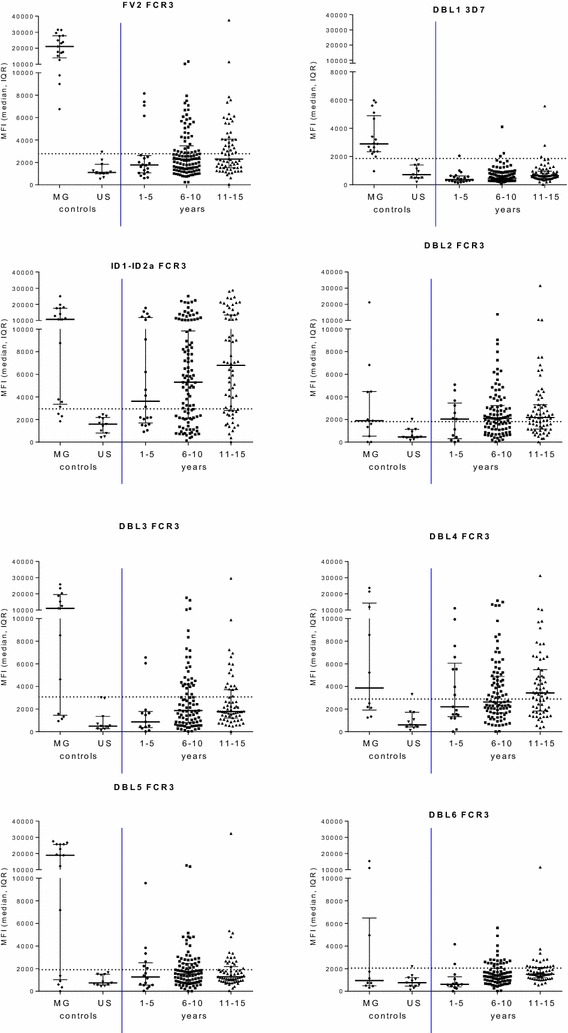
Antibody levels to VAR2CSA domains in children from Ngali II and Ntouessong rural villages. IgG levels to VAR2CSA DBL domains and full-length protein (FV2) were measured in children 0–5 years, 6–11 years and 11–15 years residing in Ngali II and Ntouessong villages. In addition, samples from 11 adult North Americans and 17 Cameroonian multigravidae were also measured as antibody-negative and -positive controls, respectively. Median MFI and Inter-Quartile Range (IQR) are plotted; *dotted line* shows cut-off for positivity

Based on immune responses to merozoite antigens, the majority of children in Ngali II and Ntouessong had IgG to malarial antigens, especially AMA1 (100%) and EBA175 (98%) (Fig. 3; Table 3). By 10 years of age, the majority of children in these villages had high IgG levels to AMA1 and EBA175 compared to younger children (Fig. 3). The proportion of children with Ab to VAR2CSA is difficult to accurately determine, since values for endemic male are usually used to establish the cut-off for Ab positivity to pregnancy-specific antigens. Using a cut-off based on North American adults, depending on the age group 56–70% children had IgG to DBL2 FCR3, 50–60% children had IgG to DBL4 and 20–44% children had IgG to FV2 (Table 3). In 11–15 year old female children, seropositivity to DBL2 FCR3 and DBL4 FCR3 was 67 and 60% respectively (Table 3; Additional file 3), demonstrating a different IgG specificity as compared to pregnant women, who have high IgG to DBL5 FCR3 and DBL3 FCR3 (Fig. 4). There were no significant differences in Ab levels to DBL domains between groups of children who were seropositive for a given antigen (Fig. 4). Children had moderate to high-level Ab to the minimal binding region ID1–ID2a (Fig. 4) with 67% of 11–15 year old girls having Ab to ID1–ID2a (Table 3). However, high Ab to ID1–ID2a were not associated with higher levels of Ab to the FV2, suggesting that Ab to ID1–ID2a do not substantially contribute to the recognition of the full-length molecule.

Since malaria prevalence was higher in 6–10 year old group of children, it was possible that they had higher IgG levels to VAR2CSA. However, IgG levels to FV2 and the DBL domains were similar across the three age groups. IgG to DBL domains from 3D7 and 7G8 strains had similar patterns (Additional file 2). The results show that Ab are most likely cross-reactive and are targeted against domains other than those recognized by pregnant women.

### Antibody levels to VAR2CSA domains in young children with severe malaria

**Fig. 5:**
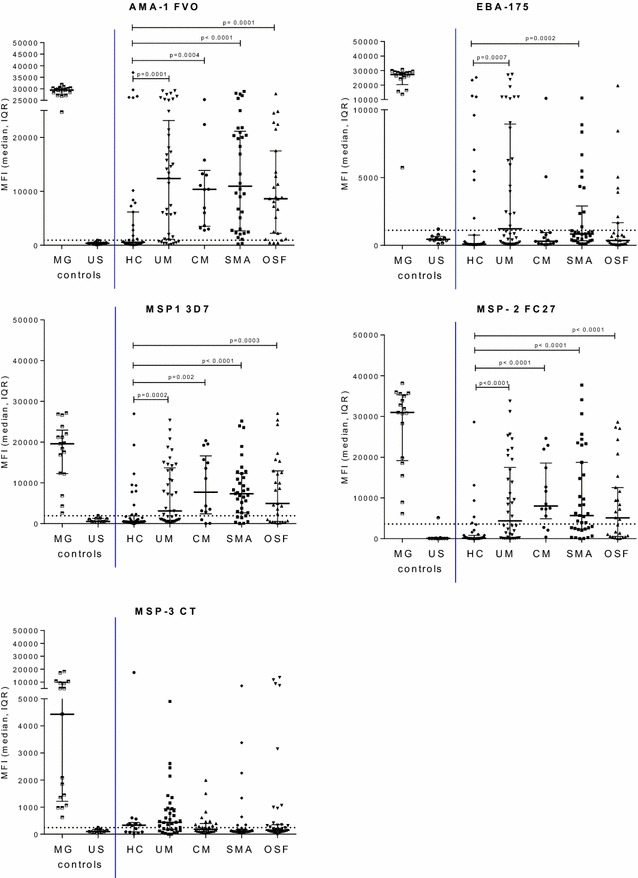
Antibody levels to merozoite antigen in children with severe malaria. IgG levels to five merozoite antigens were measured in healthy children (HC), children with uncomplicated malaria (UM), cerebral malaria (CM), severe malaria anemia (SMA) and other severe forms of malaria (OSF). In addition, samples from 11 healthy adult North American adults and 17 Cameroonian multigravidae were also measured as antibody-negative and -positive controls, respectively. Median MFI and Inter-Quartile Range (IQR) are plotted;* dotted line* shows cut-off for positivity. Antibody levels between 2 groups were compared using Mann–Whitney test

**Fig. 6:**
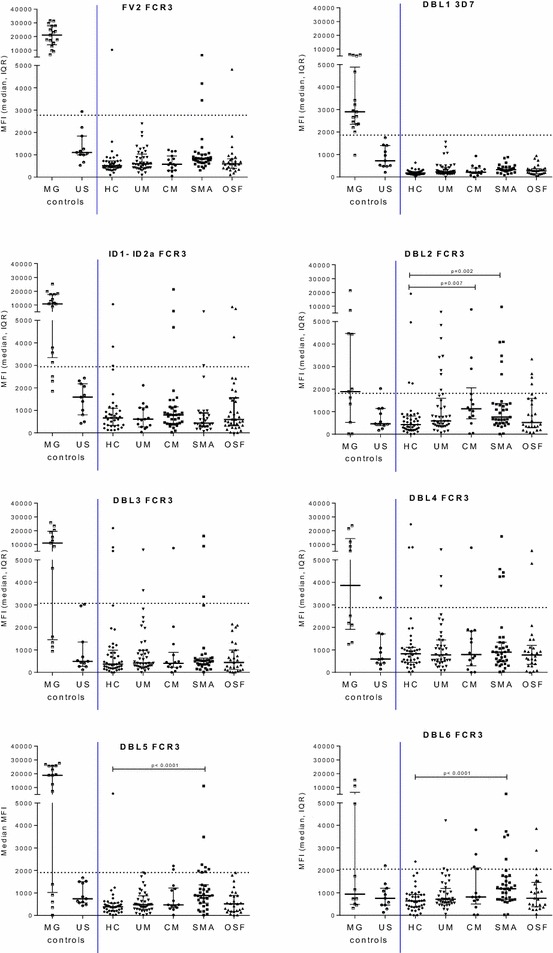
Antibody levels to VAR2CSA domains in children with mild and severe malaria. IgG levels to VAR2CSA DBL domains and full-length protein (FV2) were measured in healthy children (HC), children with uncomplicated malaria (UM), children with cerebral malaria (CM), severe malaria anemia (SMA) and other severe forms of malaria (OSF). In addition, samples from 11 healthy adult North Americans and 17 Cameroonian multigravidae were also measured as antibody-negative and -positive controls, respectively. Median MFI and Inter-Quartile Range (IQR) are plotted; *dotted line* represents cut-off for seropositivity

The characteristics of children in the severe malaria cohort (COHORT 2) enrolled at the Yaoundé Central Hospital are presented in Table 2, and more detailed clinical parameters have been previously published [39]. The parasite load was highest in children with CM with median: 570,725 parasites/µL, IQR: 996,384 parasites/µL. Hemoglobin levels were lowest in SMA group (4 ± 1.7 g/dL) (Table 2). Children with various forms of malaria had significantly higher levels of IgG to merozoite antigens compared to HC group; thus, malaria parasitaemia was associated with boosting of IgG to AMA-1, EBA-175, MSP-1 and MSP-2 (Fig. 5). It was speculated that children with hyperparasitaemia and CM might have parasite subpopulation expressing VAR2CSA and, therefore, be more likely to have IgG to VAR2CSA proteins. However, only IgG to DBL2 was significantly higher in children with CM and SMA compared to HC, while IgG to the rest of VAR2CSA domains and FV2 were rare in children with severe malaria (Fig. 6; Table 3). Similar patterns were observed for the 3D7 and 7G8 stains (Additional file 4). Thus, Ab to VAR2CSA were not more common in children with a high parasitaemia.

**Table 2:** Characteristics of children with severe malaria from Yaoundé city

	Healthy malaria negative (HMN)	Uncomplicated malaria (UM)	Cerebral malaria (CM)	Severe malaria anemia (SMA)	Others severe forms (OSF)
Total number of children	42	41	15	35	27
Average age in years (mean ± SD)	3.3 ± 2.2	3.3 ± 2.4	2.8 ± 1.7	1.6 ± 1.4	2.4 ± 1.8
Number males (percent)	20 (48%)	25 (61%)	8 (53%)	16 (46%)	12 (44%)
Malaria by blood smear positive (percent)^a^	0	100%	100%	100%	100%
Malaria parasitaemia (parasites per µL of blood, median, IQR)	–	53,225 (4098, 113,767)	570,725 (9752, 1,158,750)	23,792 (2842, 143,213)	281,168 (98,638, 885,063)
Hemoglobin levels in g/dL (mean ± SD)	12 ± 2	10 ± 2	6 ± 5	4 ± 2	8 ± 3
Anemia (number, %)^b^	14 (33%)	37 (74%)	13 (90%)	35 (100%)	26 (97%)

Additional clinical characteristics for this cohort have been reported previously by Tchinda et al. [39]

^a^Blood smears were tested by microscopy for presence of *P. falciparum*, *P. ovale*, and *P. malariae*. Only *P. falciparum* was detected

^b^Cut-offs for anemia: children 0–5 years <11 g/dL

**Table 3:** Proportion of children with antibodies to malaria antigens

	Number of samples	AMA1 3D7 (%)	DBL1 3D7	ID1–ID2a FCR3	DBL2 FCR3 (%)	DBL3 FRC3	DBL4 FCR3 (%)	DBL5 3D7	DBL5 FCR3	DBL6 FCR3 (%)	FV2 FCR3
*Ngali*-*Ntouessong village cohort (years)*											
1–5	20	100	5%	55%	70	40%	50	20%	50%	45	20%
6–10	105	100	3%	65%	62	44%	51	17%	39%	37	34%
11–15	66	99	5%	71%	56	29%	60	14%	32%	32	44%
Girls 11–15	33	97	3%	67%	67	30%	60	18%	33%	33	42%
*Severe malaria Yaoundé city cohort*											
Healthy children	42	36	5%	7%	12	10%	12	2%	5%	7	14%
Uncomplicated malaria	41	78	0	7%	20	5%	10	2%	0	7	2%
Cerebral malaria	15	100	0	0	27	13%	13	0	20%	40	0
Severe malaria anemia	35	91	0	9%	17	9%	14	3%	23%	14	11%
Other severe forms of malaria	27	85	0	7%	19	0	11	4%	0	19	4%

## Discussion

Maternal Ab to both merozoite and VAR2CSA antigens were transferred to neonates, but most Ab to FV2 and the individual DBL domains were lost by the end of the first year of life. Children began producing IgG to various DBL domains by 1–5 years of age with only a slight non-significant increase in prevalence (Table 2) and levels (Fig. 4) with increasing age. Forty-four percent of teenagers had Ab to FV2 and levels were lower than those found in MG women. The specificity of Ab responses to VAR2CSA in children appears to be different compared to MG. Children recognize DBL2, DBL3 and DBL4 and to a lesser extent DBL5, DBL6 and FV2, while MG recognize predominantly DBL3, DBL5 and FV2. Children acquire moderate to high level antibodies against the minimal binding region in of VAR2CSA (ID1–ID2a) and 67% of teenage girls had Ab to ID1–ID2a. However, naturally acquired antibodies to ID1–ID2a are not associated with absence of placental malaria at delivery [36] and do not appear to contribute substantially to recognition of full-length VAR2CSA ectodomain.

Neonates are born with passively transferred Ab from the mother during gestation. Placental transfer of antibodies from mother to the fetus is an adaptive mechanism by which deficiencies in neonate immunity are counterbalanced, providing short-term passive protection [43]. Data demonstrate that IgG to VAR2CSA DBL1, DBL2, DBL3, DBL4, DBL6 and ID1–ID2a are catabolized by 21–28 weeks of life; whereas Ab to DBL5 and the full-length FV2, that are present at higher levels at birth, persist longer with a few babies having detectable levels at 8 months of life (35–56 weeks), decline by the end of first year of life. These results are in agreement with previous findings that the amount of Ab transferred to the neonate determines the half-life [44]. In a Ghanian cohort, children had passively acquired Ab to *P. falciparum* schizont extract for a median of 14 weeks [45]. In a Nigerian cohort of neonates, maternal Ab dropped below detectable levels by 4 months of age [46].

This is the first study to establish patterns of Ab acquisition to all VAR2CSA domains, ID1–ID2a and FV2 across three age groups of 1–15 year old children. Children in Ngali II and Ntouessong villages produced low levels of IgG to VAR2CSA domains. This is in agreement with previous study demonstrating that short exposure to CSA-binding parasites during acute illness is sufficient to induce Ab responses in non-pregnant individuals [33]. High malaria prevalence and parasitaemia in these study sites makes it more likely for children to be exposed to CSA-binding parasites and, therefore, produce Ab to VAR2CSA domains.

However, data demonstrate that 6–10 year old children have the highest prevalence of malaria in Ngali/Ntouessong rural villages, but they did not have higher IgG levels compared to other age groups. In addition, hyperparasitaemic children with CM in the city did not produce more IgG to VAR2CSA as compared to the HC. IgG levels to DBL1x and DBL5ɛ were previously measured in a cohort of young Tanzanian children (38/222 with severe malaria); and about 40% of 1.5–2.5 year old children recognized DBL5ɛ [34]. The differences between two studies could be due to different malaria transmission patterns between Tanzania and Cameroon, recombinant constructs used and definitions for cut-off for seropositivity.

Although children and young adults may have Ab to VAR2CSA, the specificity, magnitude, or breadth of Ab produced in children do not protect first-time mothers from PM. Therefore, the specificity of Ab produced in children must be different from those produced by pregnant women with clinical protection from to placental malaria. This study is the first comparing the specificity of Ab to VAR2CSA between children and MG. Previous study by Gnidehou et al. tested children’s plasma samples for Ab to recombinant ID1–ID2, DBL3x and DBL5ɛ in Colombian non-pregnant individuals living in *P. falciparum* and *P. vivax* transmission area [35]. In this Cameroonian cohort of children, specificity of IgG to VAR2CSA appeared to be different from that of MG, with children more likely responding to DBL2, DBL3 and DBL4, while in MG DBL5 is most immunogenic, followed by DBL3 [36, 37, 47]. These differences in responses are important in the light of correlates of protection from PM. Correlates of protection from PM include breadth of responses to multiple VAR2CSA domains, high avidity Ab to FV2 and high levels of Ab to DBL1-2, DBL3, DBL1-3x [37, 47–50]. In addition, it is not clear how long Ab to DBL2 and DBL4 persist. Although IgG to merozoite antigens and variant surface antigens are short lived, Ab to VAR2CSA DBL5ɛ are long-lived [51, 52]. Thus, young women who have produced Ab to VAR2CSA as children may have an altered immune response upon exposure to VAR2CSA during pregnancy or placental malaria vaccine. Pre-existing IgG to VAR2CSA could limit the efficacy of VAR2CSA vaccination by eliminating vaccine immunogen, as well as interfere with the naturally acquired immunity to PM. However, results from this study demonstrate that only a small proportion of female teenagers have IgG to VAR2CSA domains and full-length ectodomain. Frequent plasma reactivity to DBL4 in children could be explained by relatively conserved nature of DBL4ε (88% sequence homology) compared to other domains [20].

This study had several limitations, including the use of recombinant VAR2CSA domains and FV2, absence of construct control (backbone of expression vector for recombinant proteins), cut-off for seropositivity was difficult to define and functionality of IgG to VAR2CSA detected in children was not tested. Data on IgG to VAR2CSA from functional assays such as inhibition of binding to CSA in children, could help define whether IgG responses observed in children are to conserved epitopes with other PfEMP1 proteins or true responses to VAR2CSA. However, results from a study by Beeson et al., who used plasma from men and children living in Papua New Guinea, Kenya and Malawi in a cell surface binding assay using both VAR2CSA-expressing and non-VAR2CSA expressing IE [33], showed that IgG-binding was specific and did not represent Ab to subpopulations of non-CSA-binding IE. At the same time, Gnidehou et al. showed that more than 50% of Colombian men who had *Plasmodium vivax* infection recognized DBL3 and DBL5, suggesting cross-reactive nature of these IgG. Thus, the role of IgG to VAR2CSA in non-pregnant individuals appear to be cross-reactive.

Interestingly, 6–15 year old children naturally acquire high IgG levels to ID1–ID2a, which is under clinical evaluation as a vaccine against PM. However, IgG to ID1–ID2a are not correlated with protection in pregnant women [36] or with IgG to the full-length VAR2CSA ectodomain. Since proportion of children with IgG to ID1–ID2a was high, but it did not translate into high proportion of children with IgG to FV2 suggests that ID1–ID2a Ab acquired during childhood do not substantially contribute to recognition of the fully-folded VAR2CSA protein on the IE surface.

## Conclusion

This study demonstrates induction of IgG to VAR2CSA domains from malaria outside pregnancy in children living in sub-Saharan Africa. Cameroonian children, including teenage girls acquire Ab to VAR2CSA domains and FV2, but Ab levels are much lower than those needed to protect women from placental infections and repertoire of Ab responses to DBL domains is different from those in pregnant women. Interestingly, children with severe malaria did not have higher Ab levels to VAR2CSA. This is the first report on naturally acquired Ab levels to ID1–ID2a, a current vaccine candidate, in a small cohort of teenage girls living in malaria high transmission settings.

## Additional files

**Additional file 1.** Antibody levels to merozoite antigens in neonates during the first year of life. Antibody levels to 5 merozoite antigens were measured in neonatal samples: n = 38 cord, n = 27 samples from 21 to 28 weeks neonates, and n = 24 samples from 35 to 56 week from neonates residing in Ngali/Ntouessong villages. In addition, 11 North American adults and 17 Cameroonian multigravidae were included as antibody-negative and -positive experimental controls. Median and interquartile ranges (IQR) are plotted, dotted line represents cut-off for seropositivity. Antibody levels between two groups were compared using Mann-Whitney test.

**Additional file 2.** IgG levels to VAR2CSA domains in 10–15 year old Cameroonian girls living in Ngali II and Ntouessong villages. IgG levels to VAR2CSA DBL domains and full-length protein (FV2) were measured in 11–15 year old girls residing in Ngali II and Ntouessong villages. DBL1 domain was from 3D7 strain and all the other proteins from FCR3 parasite strain. Median MFI and Inter-Quartile Range (IQR) are plotted.

**Additional file 3.** Antibody levels to VAR2CSA domains in children from Ngali and Ntouessong rural villages. IgG levels to VAR2CSA DBL domains and full-length protein (FV2) were measured in children 0-5 years, 6-11 years and 11-15 years residing in Ngali and Ntouessong villages. In addition, samples from 11 adult North Americans and 17 Cameroonian multigravidae were also measured as antibody-negative and -positive controls, respectively. Median MFI and Inter-Quartile Range (IQR) are plotted; dotted line shows cut-off for positivity.

**Additional file 4.** Antibody levels to VAR2CSA domains in children with mild and severe malaria. IgG levels to VAR2CSA DBL domains were measured in healthy children (HC), children with uncomplicated malaria (UM), children with cerebral malaria (CM), severe malaria anemia (SMA) and other severe forms of malaria (OSF). In addition, samples from 11 adult North Americans and 17 Cameroonian multigravidae were also measured as antibody-negative and -positive controls, respectively. Median MFI and Inter- Quartile Range (IQR) are plotted: dotted line shows cut-off for positivity.

## Abbreviations

Ab: antibodies
CM: cerebral malaria
CSA: chondroitin sulfate A
DBL: Duffy-Binding-Like
FV2: full length VAR2CSA
HC: healthy children
IE: infected erythrocytes
ID: interdomain
IMPM: Institut de Recherches Médicales et d’Etudes des Plantes Médicinales
MG: multigravida
MVDB: Malaria Vaccine Development Branch
MFI: median fluorescence intensity
OSF: others severe forms of malaria
PfEMP1: *Plasmodium falciparum* Erythrocyte Membrane Protein 1
PM: placental malaria
SMA: severe malaria anemia
UM: uncomplicated malaria

## References

[1] Kyes S, Horrocks P, Newbold C (2001). Antigenic variation at the infected red cell surface in malaria. Ann Rev Microbiol.

[2] Rowe JA, Kyes SA (2004). The role of *Plasmodium falciparum var* genes in malaria in pregnancy. Mol Microbiol.

[3] Deitsch KW, Wellems TE (1996). Membrane modifications in erythrocytes parasitized by *Plasmodium falciparum*. Mol Biochem Parasitol.

[4] Haldar K, Mohandas N (2007). Erythrocyte remodeling by malaria parasites. Curr Opin Hematol.

[5] Ferreira MU, da Silva Nunes M, Wunderlich G (2004). Antigenic diversity and immune evasion by malaria parasites. Clin Diagn Lab Immunol.

[6] Beeson JG, Brown GV (2002). Pathogenesis of *Plasmodium falciparum* malaria: the roles of parasite adhesion and antigenic variation. Cell Mol Life Sci.

[7] Newbold C, Craig A, Kyes S, Rowe A, Fernandez-Reyes D, Fagan T (1999). Cytoadherence, pathogenesis and the infected red cell surface in *Plasmodium falciparum*. Int J Parasitol.

[8] Gardner MJ, Hall N, Fung E, White O, Berriman M, Hyman RW (2002). Genome sequence of the human malaria parasite *Plasmodium falciparum*. Nature.

[9] Kyes S, Christodoulou Z, Pinches R, Kriek N, Horrocks P, Newbold C (2007). *Plasmodium falciparum var* gene expression is developmentally controlled at the level of RNA polymerase II-mediated transcription initiation. Mol Microbiol.

[10] Scherf A, Hernandez-Rivas R, Buffet P, Bottius E, Benatar C (1998). Antigenic variation in malaria: in situ switching, relaxed and mutually exclusive transcription of var genes during intra-erythrocytic development in *Plasmodium falciparum*. EMBO J.

[11] Chen Q, Fernandez V, Sundstrom A, Schlichtherle M, Datta S, Hagblom P (1998). Developmental selection of var gene expression in *Plasmodium falciparum*. Nature.

[12] Salanti A, Dahlback M, Turner L, Nielsen MA, Barfod L, Magistrado P (2004). Evidence for the involvement of VAR2CSA in pregnancy-associated malaria. J Exp Med.

[13] Tuikue Ndam NG, Salanti A, Bertin G, Dahlback M, Fievet N (2005). High level of var2csa transcription by *Plasmodium falciparum* isolated from the placenta. J Infect Dis.

[14] Rovira-Vallbona E, Dobano C, Bardaji A, Cistero P, Romagosa C, Serra-Casas E (2011). Transcription of var genes other than var2csa in *Plasmodium falciparum* parasites infecting Mozambican pregnant women. J Infect Dis.

[15] Bancells C, Deitsch KW (2013). A molecular switch in the efficiency of translation reinitiation controls expression of var2csa, a gene implicated in pregnancy-associated malaria. Mol Microbiol.

[16] Amulic B, Salanti A, Lavstsen T, Nielsen MA, Deitsch KW (2009). An upstream open reading frame controls translation of var2csa, a gene implicated in placental malaria. PLoS Pathog.

[17] Srivastava A, Gangnard S, Round A, Dechavanne S, Juillerat A, Raynal B, Faure G (2010). Full-length extracellular region of the var2CSA variant of PfEMP1 is required for specific, high-affinity binding to CSA. Proc Natl Acad Sci USA.

[18] Muthusamy A, Achur RN, Bhavanandan VP, Fouda GG, Taylor DW, Gowda DC (2004). *Plasmodium falciparum*-infected erythrocytes adhere both in the intervillous space and on the villous surface of human placenta by binding to the low-sulfated chondroitin sulfate proteoglycan receptor. Am J Pathol.

[19] Pasternak ND, Dzikowski R (2009). PfEMP1: an antigen that plays a key role in the pathogenicity and immune evasion of the malaria parasite *Plasmodium falciparum*. Int J Biochem Cell Biol.

[20] Bockhorst J, Lu F, Janes JH, Keebler J, Gamain B, Awadalla P, Su XZ, Samudrala R (2007). Structural polymorphism and diversifying selection on the pregnancy malaria vaccine candidate VAR2CSA. Mol Biochem Parasitol.

[21] Clausen TM, Christoffersen S, Dahlback M, Langkilde AE, Jensen KE, Resende M (2012). Structural and functional insight into how the *Plasmodium falciparum* VAR2CSA protein mediates binding to chondroitin sulfate A in placental malaria. J Biol Chem.

[22] Srivastava A, Gangnard S, Dechavanne S, Amirat F, Lewit Bentley A (2011). Var2CSA minimal CSA binding region is located within the N-terminal region. PLoS ONE.

[23] McGregor IA (1984). Epidemiology, malaria and pregnancy. Am J Trop Med Hyg.

[24] Brabin BJ, Premji Z, Verhoeff F (2001). An analysis of anemia and child mortality. J Nutr..

[25] O’Neil-Dunne I, Achur RN, Agbor-Enoh ST, Valiyaveettil M, Naik RS (2001). Gravidity-dependent production of antibodies that inhibit binding of *Plasmodium falciparum*-infected erythrocytes to placental chondroitin sulfate proteoglycan during pregnancy. Infect Immun.

[26] Fried M, Nosten F, Brockman A, Brabin BJ, Duffy PE (1998). Maternal antibodies block malaria. Nature.

[27] Barfod L, Dobrilovic T, Magistrado P, Khunrae P, Viwami F, Bruun J, Dahlback M (2010). Chondroitin sulfate A-adhering *Plasmodium falciparum*-infected erythrocytes express functionally important antibody epitopes shared by multiple variants. J Immunol..

[28] Feng G, Aitken E, Yosaatmadja F, Kalilani L, Meshnick SR, Jaworowski A (2009). Antibodies to variant surface antigens of *Plasmodium falciparum*-infected erythrocytes are associated with protection from treatment failure and the development of anemia in pregnancy. J Infect Dis.

[29] Duffy PE, Fried M (2003). Antibodies that inhibit *Plasmodium falciparum* adhesion to chondroitin sulfate A are associated with increased birth weight and the gestational age of newborns. Infect Immun.

[30] Staalsoe T, Megnekou R, Fievet N, Ricke CH, Zornig HD, Leke R (2001). Acquisition and decay of antibodies to pregnancy-associated variant antigens on the surface of *Plasmodium falciparum*-infected erythrocytes that protect against placental parasitemia. J Infect Dis.

[31] Nielsen MA, Resende M, de Jongh WA, Ditlev SB, Mordmuller B, Houard S (2015). The influence of sub-unit composition and expression system on the functional antibody response in the development of a VAR2CSA based *Plasmodium falciparum* placental malaria vaccine. PLoS ONE.

[32] Thrane S, Janitzek CM, Agerbaek MO, Ditlev SB, Resende M, Nielsen MA (2015). A novel virus-like particle based vaccine platform displaying the placental malaria antigen VAR2CSA. PLoS ONE.

[33] Beeson JG, Ndungu F, Persson KE, Chesson JM, Kelly GL (2007). Antibodies among men and children to placental-binding *Plasmodium falciparum*-infected erythrocytes that express var2csa. Am J Trop Med Hyg.

[34] Oleinikov AV, Voronkova VV, Frye IT, Amos E, Morrison R, Fried M, Duffy PE (2012). A plasma survey using 38 PfEMP1 domains reveals frequent recognition of the *Plasmodium falciparum* antigen VAR2CSA among young Tanzanian children. PLoS ONE.

[35] Gnidehou S, Doritchamou J, Arango EM, Cabrera A, Arroyo MI, Kain KC (2014). Functional antibodies against VAR2CSA in nonpregnant populations from colombia exposed to *Plasmodium falciparum* and *Plasmodium vivax*. Infect Immun.

[36] Babakhanyan A, Leke RG, Salanti A, Bobbili N, Gwanmesia P, Leke RJ (2014). The antibody response of pregnant Cameroonian women to VAR2CSA ID1–ID2a, a small recombinant protein containing the CSA-binding site. PLoS ONE.

[37] Babakhanyan A, Fang R, Wey A, Salanti A, Sama G, Efundem C, Leke RJ (2015). Comparison of the specificity of antibodies to VAR2CSA in Cameroonian multigravidae with and without placental malaria: a retrospective case-control study. Malar J..

[38] Babakhanyan A, Tutterrow YL, Bobbili N, Salanti A, Wey A (2016). Influence of intermittent preventive treatment on antibodies to VAR2CSA in pregnant Cameroonian women. Am J Trop Med Hyg.

[39] Tchinda VH, Tadem AD, Tako EA, Tene G, Fogako J, Nyonglema P (2007). Severe malaria in Cameroonian children: correlation between plasma levels of three soluble inducible adhesion molecules and TNF-alpha. Acta Trop.

[40] Louis JP, Trebucq A, Gelas H, Fondjo E, Manga L, Toto JC, Carnevale P (1992). [Malaria in Yaounde (Cameroon). Cost and antivectorial control at the family level](in French). Bull Soc Pathol Exot.

[41] Manga L, Traore O, Cot M, Mooh E, Carnevale P (1993). [Malaria in the village of Yaounde (Cameroon). 3. Parasitological study in 2 central districts](in French). Bull Soc Pathol Exot.

[42] Fouda GG, Leke RF, Long C, Druilhe P, Zhou A, Taylor DW, Johnson AH (2006). Multiplex assay for simultaneous measurement of antibodies to multiple *Plasmodium falciparum* antigens. Clin Vaccine Immunol.

[43] Palmeira P, Quinello C, Silveira-Lessa AL, Zago CA, Carneiro-Sampaio M (2012). IgG placental transfer in healthy and pathological pregnancies. Clin Dev Immunol..

[44] de Moraes-Pinto I, Hart CA (1997). Transplacental antibody transfer and neonatal immunity. Br J Hosp Med.

[45] Riley EM, Wagner GE, Ofori MF, Wheeler JG, Akanmori BD, Tetteh K (2000). Lack of association between maternal antibody and protection of African infants from malaria infection. Infect Immun.

[46] Achidi EA, Perlmann H, Salimonu LS, Perlmann P, Walker O, Asuzu MC (1995). A longitudinal study of seroreactivities to *Plasmodium falciparum* antigens in Nigerian infants during their first year of life. Acta Trop.

[47] Tutterrow YL, Avril M, Singh K, Long CA, Leke RJ, Sama G, Salanti A (2012). High levels of antibodies to multiple domains and strains of VAR2CSA correlate with the absence of placental malaria in Cameroonian women living in an area of high *Plasmodium falciparum* transmission. Infect Immun.

[48] Tutterrow YL, Salanti A, Avril M, Smith JD, Pagano IS, Ako S (2012). High avidity antibodies to full-length VAR2CSA correlate with absence of placental malaria. PLoS ONE.

[49] Ndam NT, Denoeud-Ndam L, Doritchamou J, Viwami F, Salanti A, Nielsen MA (2015). Protective antibodies against placental malaria and poor outcomes during pregnancy, Benin. Emerg Infect Dis..

[50] Dechavanne S, Srivastava A, Gangnard S, Nunes-Silva S, Dechavanne C (2015). Parity-dependent recognition of DBL1X-3X suggests an important role of the VAR2CSA high-affinity CSA-binding region in the development of the humoral response against placental malaria. Infect Immun.

[51] Kinyanjui SM, Bull P, Newbold CI, Marsh K (2003). Kinetics of antibody responses to *Plasmodium falciparum*-infected erythrocyte variant surface antigens. J Infect Dis.

[52] Fowkes FJ, McGready R, Cross NJ, Hommel M, Simpson JA, Elliott SR (2012). New insights into acquisition, boosting, and longevity of immunity to malaria in pregnant women. J Infect Dis.

