# Supplementary figures and images for: Antibody responses to the full-length VAR2CSA and its DBL domains in Cameroonian children and teenagers
Source: Malar J. 2016 Nov 4;15:532. doi: 10.1186/s12936-016-1585-y (PMC5097422; doi:10.1186/s12936-016-1585-y)

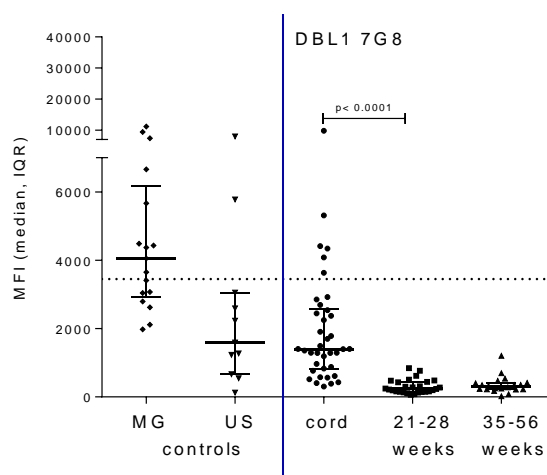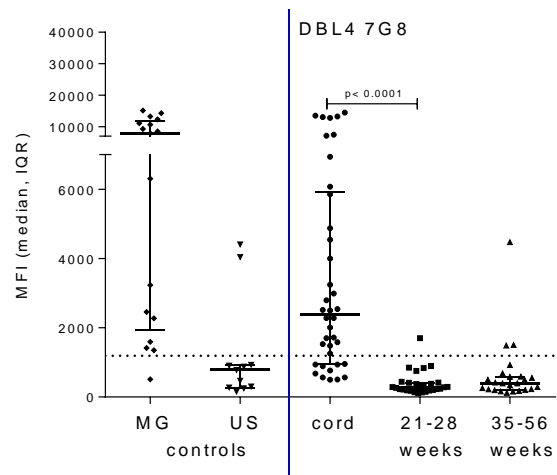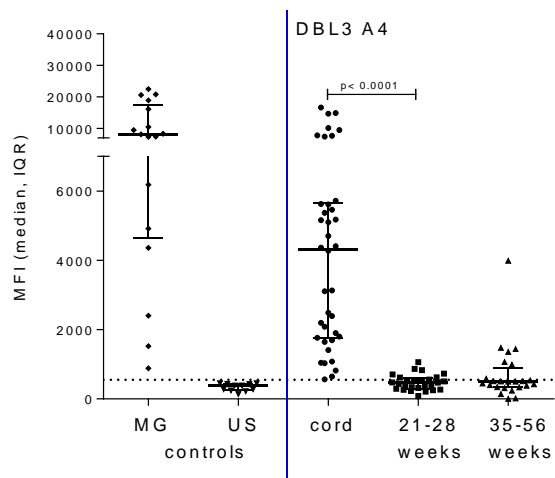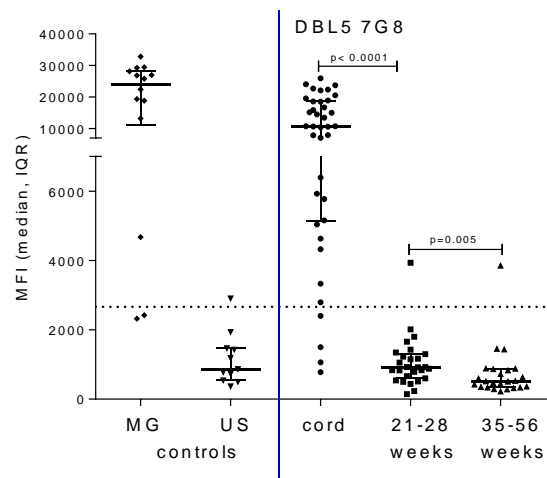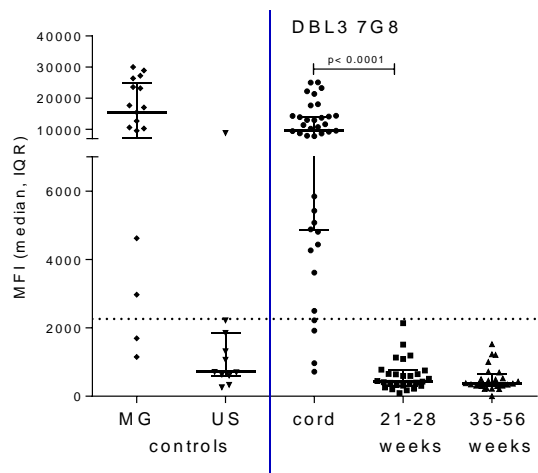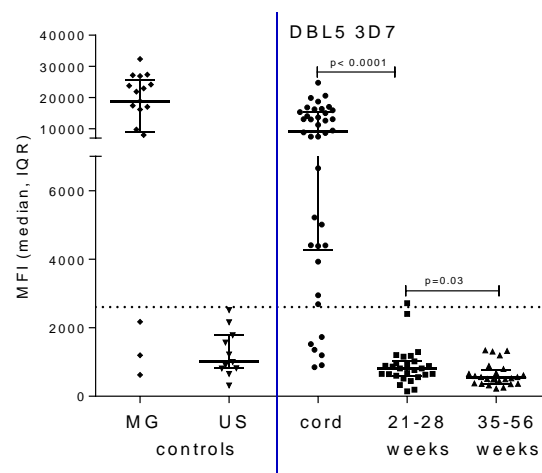

Supplement: Supplementary file 1 — Additional file 1. Antibody levels to merozoite antigens in neonates during the first year of life. Antibody levels to 5 merozoite antigens were measured in neonatal samples: n = 38 cord, n = 27 samples from 21 to 28 weeks neonates, and n = 24 samples from 35 to 56 week from neonates residing in Ngali/Ntouessong villages. In addition, 11 North American adults and 17 Cameroonian multigravidae were included as antibody-negative and -positive experimental controls. Median and interquartile ranges (IQR) are plotted, dotted line represents cut-off for seropositivity. Antibody levels between two groups were compared using Mann-Whitney test. [file 12936_2016_1585_MOESM1_ESM.pdf]

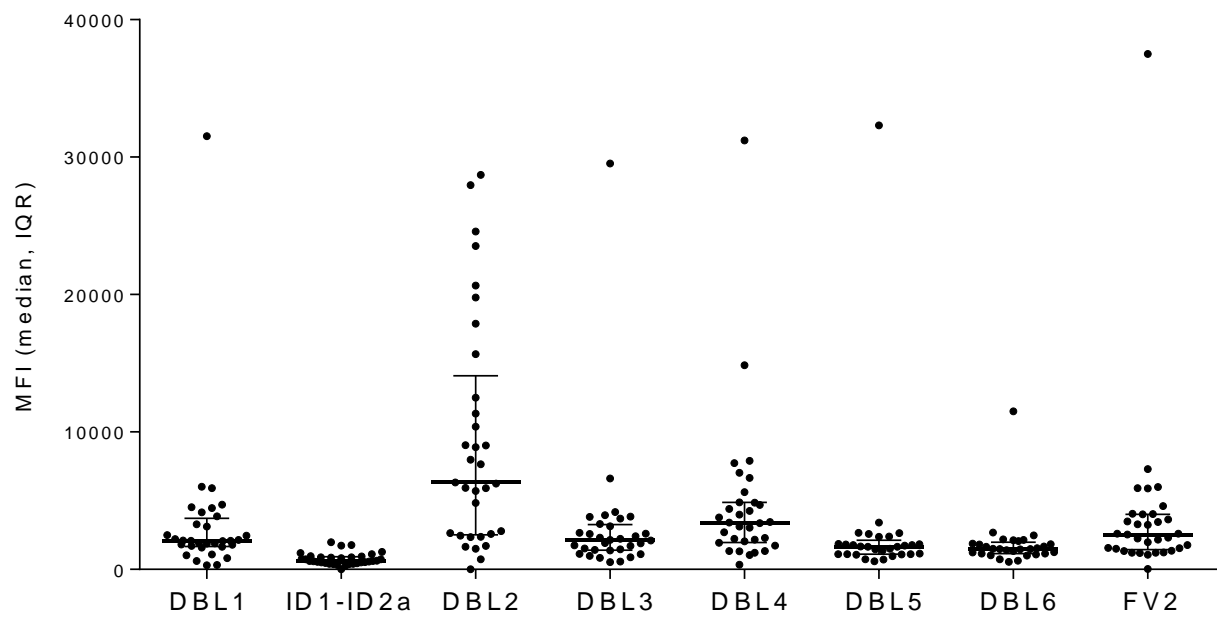

Supplement: Supplementary file 2 — Additional file 2. IgG levels to VAR2CSA domains in 10–15 year old Cameroonian girls living in Ngali II and Ntouessong villages. IgG levels to VAR2CSA DBL domains and full-length protein (FV2) were measured in 11–15 year old girls residing in Ngali II and Ntouessong villages. DBL1 domain was from 3D7 strain and all the other proteins from FCR3 parasite strain. Median MFI and Inter-Quartile Range (IQR) are plotted. [file 12936_2016_1585_MOESM2_ESM.pdf]

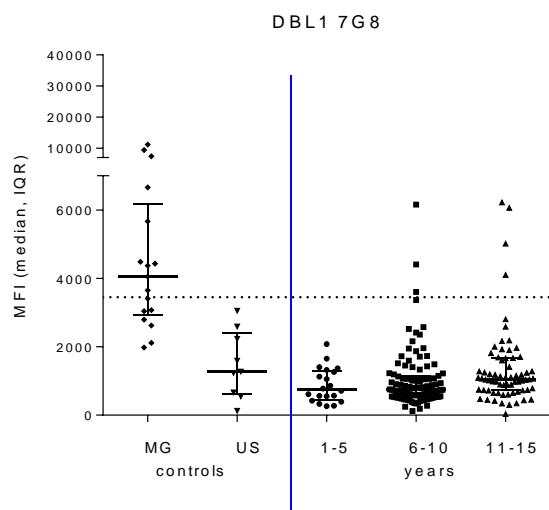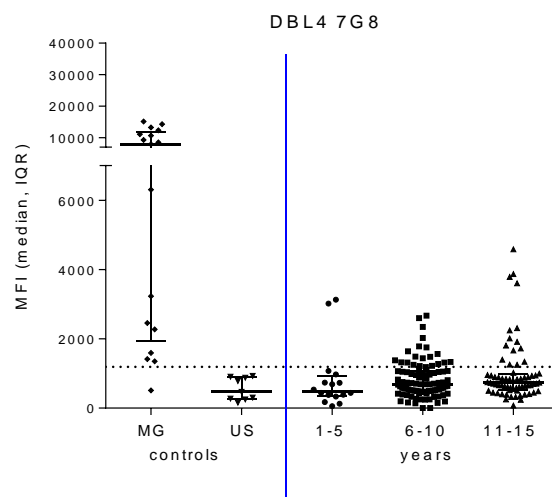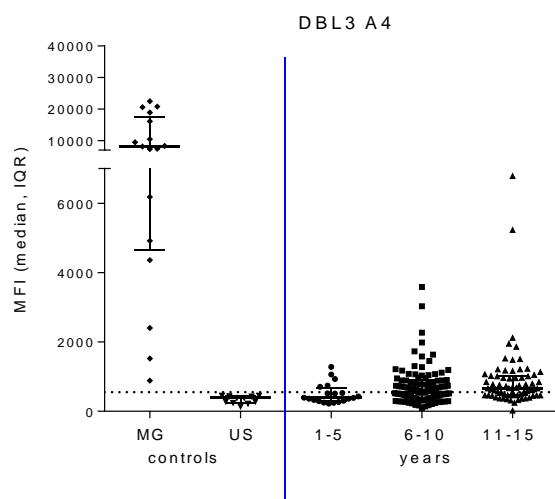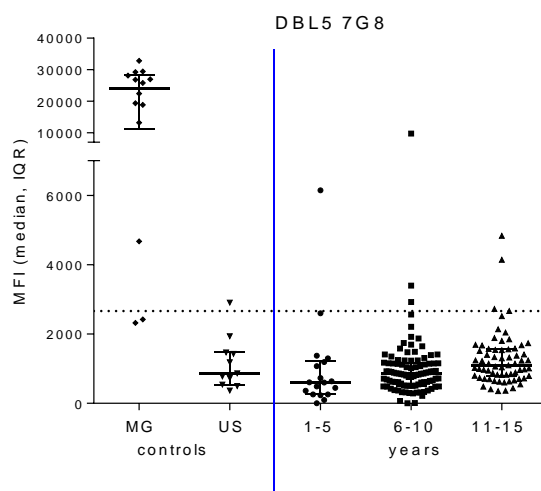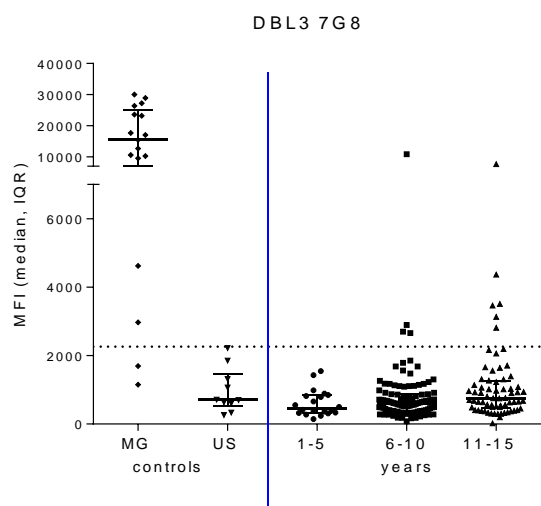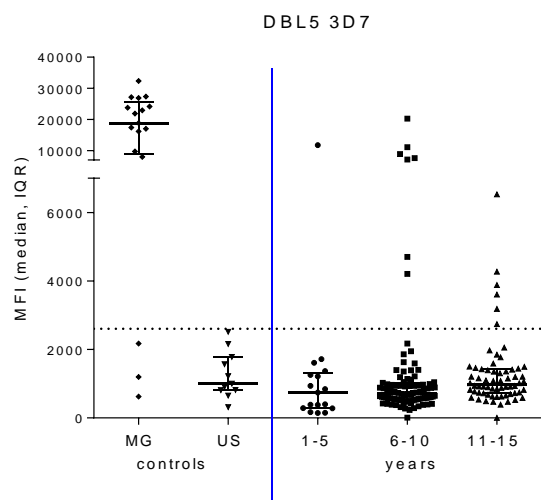

Supplement: Supplementary file 3 — Additional file 3. Antibody levels to VAR2CSA domains in children from Ngali and Ntouessong rural villages. IgG levels to VAR2CSA DBL domains and full-length protein (FV2) were measured in children 0-5 years, 6-11 years and 11-15 years residing in Ngali and Ntouessong villages. In addition, samples from 11 adult North Americans and 17 Cameroonian multigravidae were also measured as antibody-negative and -positive controls, respectively. Median MFI and Inter-Quartile Range (IQR) are plotted; dotted line shows cut-off for positivity. [file 12936_2016_1585_MOESM3_ESM.pdf]

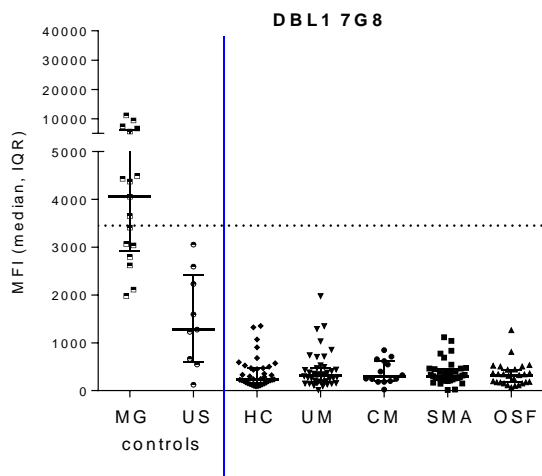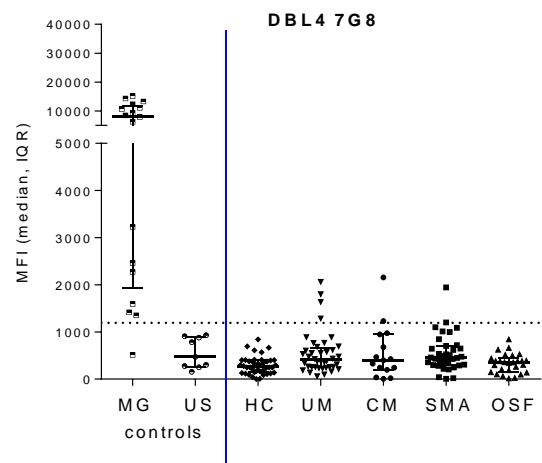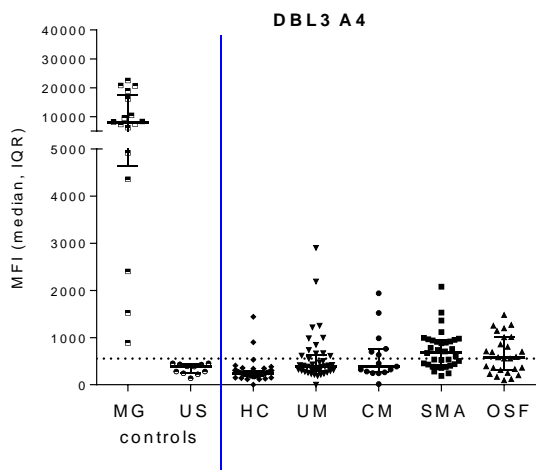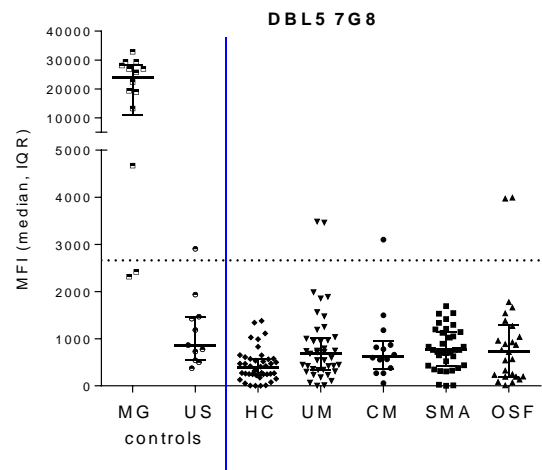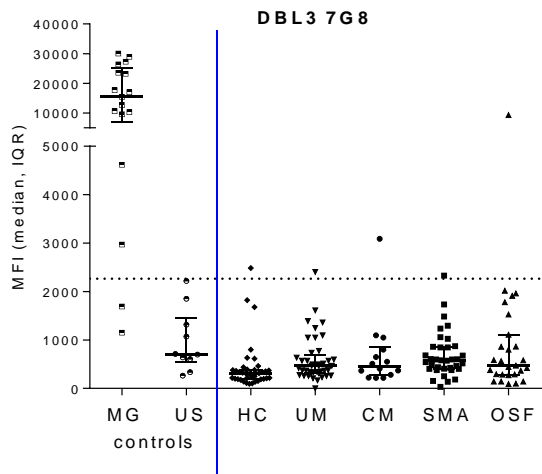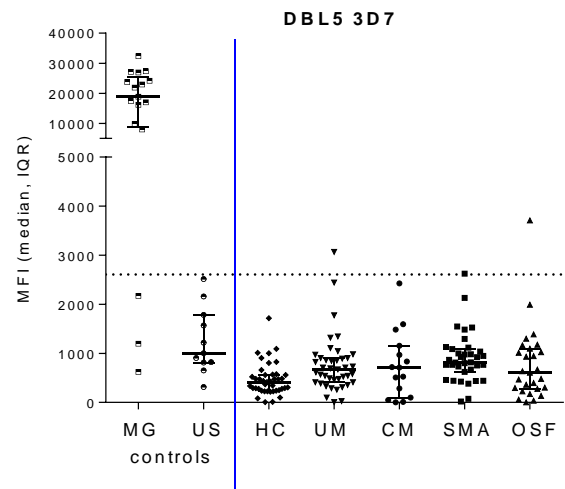

Supplement: Supplementary file 4 — Additional file 4. Antibody levels to VAR2CSA domains in children with mild and severe malaria. IgG levels to VAR2CSA DBL domains were measured in healthy children (HC), children with uncomplicated malaria (UM), children with cerebral malaria (CM), severe malaria anemia (SMA) and other severe forms of malaria (OSF). In addition, samples from 11 adult North Americans and 17 Cameroonian multigravidae were also measured as antibody-negative and -positive controls, respectively. Median MFI and Inter- Quartile Range (IQR) are plotted: dotted line shows cut-off for positivity. [file 12936_2016_1585_MOESM4_ESM.pdf]
